# Combining space use with diet data to investigate foraging tactics of black bears in response to the pulsed availability of migratory caribou calves

**DOI:** 10.1371/journal.pone.0346054

**Published:** 2026-04-03

**Authors:** Linda Nowack, Michaël Bonin, Martin Leclerc, Candice Michelot, Christian Dussault, Joëlle Taillon, John Pisapio, Steeve D. Côté

**Affiliations:** 1 Département de biologie, Caribou Ungava, Université Laval, Québec, Québec, Canada; 2 Direction principale de l’expertise sur la faune terrestre, Ministère de l’Environnement, de la Lutte contre les changements climatiques, de la Faune et des Parcs, Québec, Québec, Canada; 3 Department of Fisheries Forestry and Agriculture, Government of Newfoundland and Labrador, Port Hope Simpson, Newfoundland, Canada; University of Bucharest, ROMANIA

## Abstract

Migratory caribou (*Rangifer tarandus*) is a key component of the arctic food web. Female caribou typically gather in late spring to give birth, creating a predictable, ephemeral resource pulse that can influence consumer behavior. As an omnivore, the black bear (*Ursus americanus*) predates on caribou neonates during calving when they are most vulnerable and occur at high densities. We studied black bears in northern Québec and Labrador interacting with the Rivière-aux-Feuilles (RAFH) and Rivière-George (RGH) migratory caribou herds. Our aim was to assess the spatial response of bears to caribou distribution during calving by investigating the potential correlation between bear movements and their relative trophic position. We expected bears with higher trophic positions to adopt behaviors favoring caribou encounters during the caribou calving period. We used GPS telemetry data from 40 bears and 319 female caribou between 2012 and 2019 to calculate movement metrics and home ranges, and to assess seasonal variation in movement patterns and shared space use between bears and caribou. We then analyzed the relationships between space use data and relative trophic position of bears obtained from stable isotope analysis of blood serum. The relative trophic position of black bears differed between study sites, with individuals in northern Québec (RAFH) exhibiting higher trophic positions than those in Labrador (RGH). Relative trophic positions varied strongly among individuals, indicating substantial individual differences in foraging strategies. Greater proportional overlap of bear ranges with caribou calving ground was associated with higher trophic positions, while higher use of caribou-preferred habitat by bears surprisingly correlated with lower relative trophic positions. The absence of a clear link between movement metrics and trophic position may reflect the inherently opportunistic foraging behavior of black bears. Additional research at the individual level is needed to gain deeper insight into black bear foraging strategies in response to pulsed resources.

## Introduction

Variation in resource availability and distribution can influence animal movements to access food sources [1,2]. A particular case of variation in resource availability is pulsed resources [3], which are defined as a sudden peak of a resource over a brief period followed by a steep decrease in abundance [4]. Pulsed resources are remarkably diverse and include events such as mast production [5], insect irruptions [6], large input of animal carcasses [7] or synchronous spawning events [8]. Such pulses are often recurring in time and space, making them highly predictable for consumers. Temporary increases in resource abundance may translate into behavioral as well as numerical responses by consumers, leading to population growth [6], decreased home range sizes [9] or directed movements towards the pulsed resource [10,11]. Generalists are more opportunistic foragers and therefore often exhibit more pronounced responses to a pulse resource than specialists [4,12]. Because resource pulses are ephemeral events with generally a long inter-pulse interval, generalist consumers can switch back to an alternate diet after the pulse vanishes [4,12,13].

Opportunistic omnivores are generalists that can feed across multiple trophic levels [14–16]. They often face trade-offs between using abundant but low-quality food (vegetation) or allocating high search efforts to access less available but high-quality food sources (animal protein) [17–19]. Encounters between opportunistic predators and their prey are assumed to be incidental rather than intentional [20]. Foraging decisions, however, can change in relation to the availability of local resources, and omnivores may switch from a rather vegetarian or opportunistic foraging tactic to a more active hunting mode when prey are temporally abundant, spatially aggregated [21] or more vulnerable than usual [19]. Generalist species occupy a broad foraging niche, and individuals generally exhibit high behavioral plasticity which enables them to exploit pulses and other heterogenous and dispersed resources [22].

Plasticity in movement and space use tactics in generalist consumers is key to cope with fluctuations in food availability [23] and can serve as an indicator of which foraging tactic is used [24,25] or which diet is preferred [26]. In general, hunting and increased meat consumption are associated with higher search effort due to the mobility of prey pushing consumers to move over larger ranges compared to when foraging on immobile food sources, e.g., vegetation [27]. Speed of movement is also a determining factor of foraging tactic and was reported to be higher in more carnivorous species [27] or certain individuals within a population with higher animal protein intake [26]. Such patterns likely arise because pursuing mobile prey requires faster and more intense movements than feeding on stationary vegetation. Despite the general observation of higher movement rates in carnivorous species, several other behaviors may also influence a predator’s efficiency, resulting in a variety of potential movement strategies. Hunting success for instance, can depend on habitat selection [28], which could increase the possibility to encounter a certain prey or might affect its vulnerability towards predation [19]. The spatio-temporal distribution and availability of resources also influence foraging-related movements of consumers and the search intensity which is needed to exploit the resource. When prey are highly aggregated, slower movements with high turning rate are likely to be used [29], whereas faster and straighter movements are expected to be performed to increase efficiency while foraging between food patches and facing higher variability in prey spatial distribution [30,31]. Additionally, the predictability of the availability of a resource, as it is the case for a pulsed resource, could enable an animal to navigate directly towards a destination with orientated and straight movements [32]. Space use models of a food-maximizing tactic suggest that home range size is also affected by food quality, availability, and predictability [33]. In the case of a pulse of a high-quality food resource accessible within a limited range, home range sizes of consumers should decrease [9,34] because energy requirements can be fulfilled with a lower search effort. Decreases in home range size with increases in food abundance and predictability have been shown for brown bear (*Ursus arctos*) populations across North America [33].

The American black bear (*Ursus americanus*) is a large, solitary and wide-ranging omnivore with the ability to undertake long-distance movements, especially when food is scarce [35,36]. Although black bears show highly flexible foraging tactics, their diet is mainly based on vegetation across most of its range in North America, and consumption of animal prey is highly variable across populations and even individuals [37–39]. While they occasionally prey on adult ungulates (e.g., deer *Odocoileus sp.*, moose *Alces alces*, caribou *Rangifer sp*.), black bears predominantly target neonates during the calving season when they are less mobile and easier to catch [12,40,41]. Yet, black bear predation on ungulate neonates may result from active search [19] or opportunistic encounters [17], depending on environmental conditions. Rayl et al. [19] and Edwards et al. [26] reported active foraging tactics in bears, indicating that black bears select areas with a higher probability of encountering caribou and greater hunting success, whereas higher movement rates in grizzly bears were linked to increased meat intake. Opportunistic foraging, on the other hand, has been suggested for black bear populations preying on forest-dwelling caribou, with bears primarily selecting areas with high plant biomass [17]. Both foraging tactics (active vs. opportunistic predation) have been shown to occur within a bear population, and factors such as sex, body size and individual experience of bears could determine an individual’s foraging tactic [26]. In species with sexual dimorphism such as black bears, males are generally expected to have a higher relative trophic position than females [26,42], suggesting that sex might explain part of the behavioral differences in foraging tactics within a population [43]. Prey density and vulnerability may also influence foraging tactics of predators, especially for omnivorous and generalist species [19]. For instance, in the boreal forest, black bears have a low probability of encountering boreal caribou (*Rangifer tarandus caribou*) calves and adopt an opportunistic foraging tactic by using habitats with high vegetation cover that are more often used by female caribou during calving [17]. In Newfoundland, on the other hand, where female caribou aggregate on calving grounds to give birth synchronously, active predation by black bears on caribou calves has been reported [19,44].

Migratory caribou are a key component of northern food webs and aggregate each spring in traditional calving grounds to give birth synchronously [45,46]. Caribou neonates are particularly vulnerable to predation during the first weeks of life [47–49] and their occurrence can be considered as a predictable pulse resource in space and time that may temporarily influence the distribution of predators, including black bears [12,50]. Because migratory caribou herds in northern Québec and Labrador have experienced large declines in abundance in recent decades [51,52], a better understanding of the response of predators to the pulse of caribou calves could shed light on their potential impact on caribou population dynamics.

We expected bears with a higher relative trophic position to perform movement and space use behaviors indicative of an active hunting tactic during the caribou calving period, likely resulting in increased encounter rates and hunting success, compared to bears with a lower relative trophic position. To describe black bear foraging tactics, we linked the relative trophic position of individual bears with their space use data recorded with GPS collars. We expected bears with a higher relative trophic position to take advantage of caribou neonates during calving and therefore to forage near the calving grounds. We also predicted that bears with higher relative trophic positions would adjust their foraging tactics and display movement and space-use patterns to access caribou neonates more effectively by exhibiting faster movements, larger home ranges, and lower turning rates compared to bears using immobile food sources (i.e., foraging mainly on vegetation) and having lower relative trophic positions. Alternatively, bears with higher relative trophic position could also adjust their movement and space use to the high density of caribou calves by foraging within small ranges centered on the calving ground, with lower pace and increased turning rates. We predicted that in both cases, bears with higher relative trophic positions should show a more pronounced overlap with the core areas of the caribou calving ground and should use habitat types and elevations where caribou are more likely to occur compared to bears with lower relative trophic positions.

## Materials and methods

### Study area

**Fig 1:**
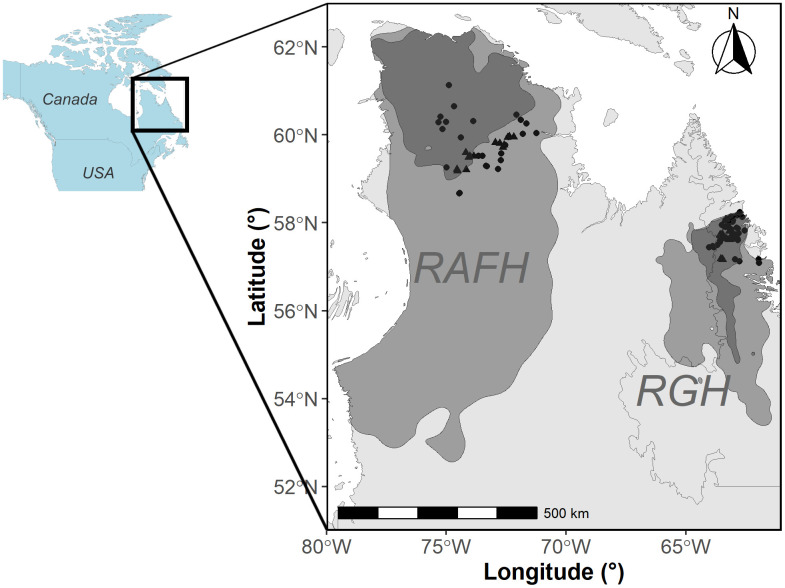
Study area with the capture locations of male and female black bears on the Rivière-aux-Feuilles and Rivière-George migratory caribou ranges in northern Québec and Labrador. RAFH = Rivière-aux-Feuilles herd; RGH = Rivière-George herd; Dots indicate the capture locations of males and triangles are the capture locations of female black bears. The grey areas represent the annual caribou range, while the darker grey areas show the caribou calving ground, both estimated using 95% kernel densities of telemetry locations collected from 2012 to 2019. Basemap data © Government of Canada, Geospatial Information Directorate. Contains information licensed under the Open Government Licence – Canada.

**Fig 2:**
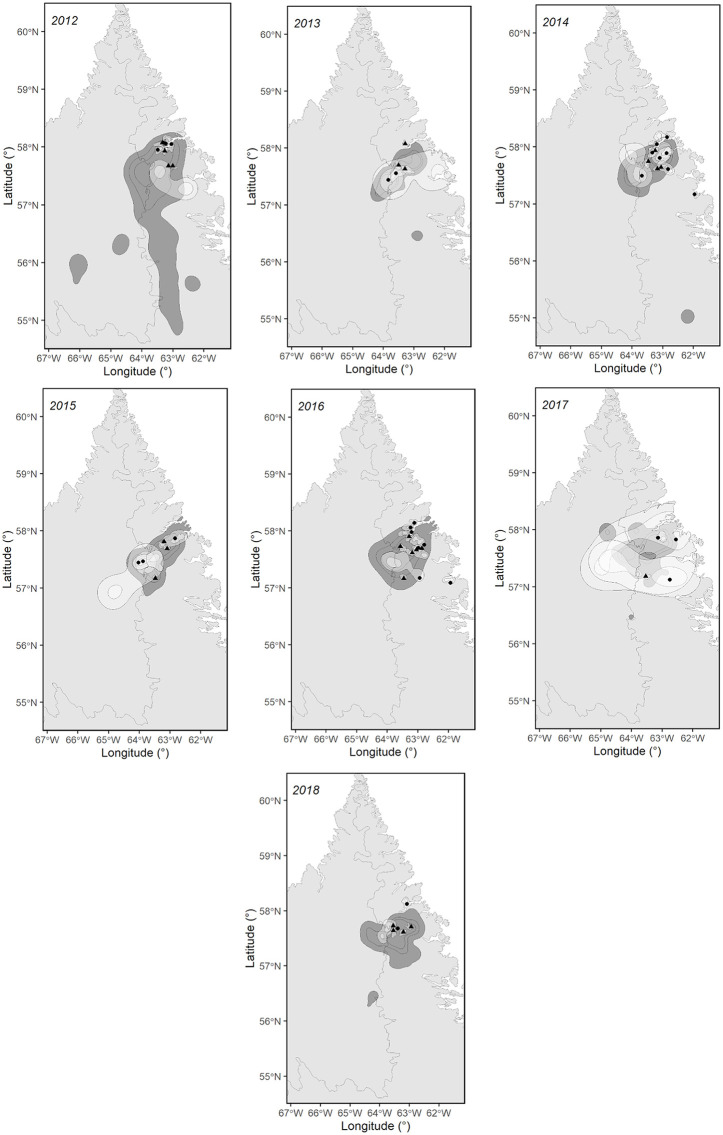
Annual caribou calving grounds of the Rivière-George herd and ranges of black bears in northern Québec and Labrador. The dark grey areas represent the annual caribou calving grounds (95%, 75%, and 50% Kernels), while the light grey areas show the utilized ranges (95% Kernel) and core areas of black bears (50% Kernel) during the caribou calving period, based on telemetry data collected from 2014 to 2018. Black dots indicate male bear capture locations, and triangles represent female bear capture locations. Basemap data © Government of Canada, Geospatial Information Directorate. Contains information licensed under the Open Government Licence – Canada.

**Fig 3:**
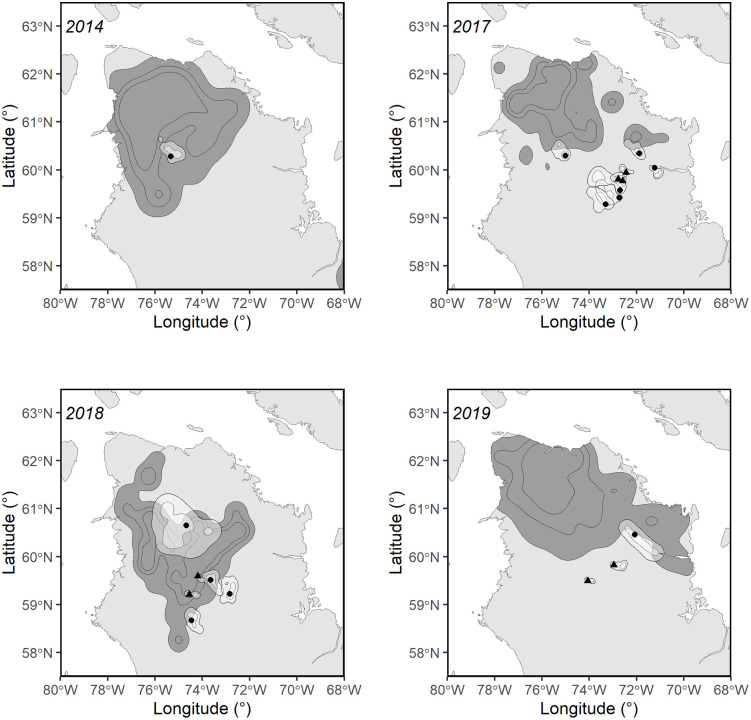
Annual caribou calving grounds of the Rivière-aux-Feuilles herd and ranges of black bears in northern Québec. The dark grey areas represent the annual caribou calving grounds (95%, 75%, and 50% Kernels), while the light grey areas show the utilized ranges (95% Kernel) and core areas of black bears (50% Kernel) during the caribou calving period based on telemetry data collected from 2014 to 2019. Black dots indicate male bear capture locations, and triangles represent female bear capture locations. Basemap data © Government of Canada, Geospatial Information Directorate. Contains information licensed under the Open Government Licence – Canada.

We studied black bears in northern Québec and Labrador, Canada (Fig 1), where they are sympatric with two migratory caribou herds, the Rivière-aux-Feuilles (RAFH) (Fig 2) and Rivière-George (RGH) (Fig 3). Despite the absence of survey data, field observations and mentions by local people suggest that black bear population size within the range of the RGH increased between 1970 and 1989 and the population is currently well established with seasonal high-density zones [45]. On the RAFH range, the black bear population has established during recent decades (MELCCFP; Caribou Ungava, unpublished data), expanded to the northern fringe of the Ungava Peninsula, and is now interacting with caribou. Both migratory caribou herds, in northern Québec and Labrador, have shown large population declines during the last decades [53,54]. The RAFH showed a decrease from > 600,000 caribou at the onset of the 2000s [54] to about 199,000 (± 16,000) individuals in 2016. The RGH went from ca. 800,000 (± 104,000) individuals in 1993 [55] to < 7,200 (± 432) individuals in 2022 (MELCCFP, unpublished data), representing a decrease of 99% attributed to several limiting factors and threats.

The calving ground of the migratory caribou of the RGH is located on the plateaus east of the Québec-Labrador Peninsula (57°N, 65°W). Female caribou from the RAFH calve further north-west in the Ungava Peninsula (61°N, 74°W; Fig 1). The ranges of the calving grounds are dependent on population size and can vary accordingly [52]. Between 2012 and 2019, calving ground areas, estimated with a 95% Kernel using telemetry data of female caribou during the calving period, averaged 68,802 km^2^ for the RAFH and 14,966 km^2^ for the RGH (S1 Table). The study areas are located in low arctic and typical arctic tundra ecosystem [56] as well as in substantial montane areas in Labrador. Both herd ranges experience low levels of human footprints [57] and a sub-arctic or arctic climate [58].

### Capture and monitoring of caribou and bears

We captured female caribou (≥ 1.5 years) and black bears (> 2 years) on both study sites between 2012 and 2019. We conducted all captures from a helicopter using either a net gun for caribou or a dart gun for bears. We used physical restrain to manipulate caribou. We equipped 254 female caribou from the RAFH (641 caribou-years) and 205 from the RGH (475 caribou-years; S1Table) with GPS collars (Vectronic, Aerospace, Berlin, Germany) programmed to record positions every 13 hours. We monitored a total of 35 bears with GPS collars (model Vertex Lite and Vertex Plus from Vectronic, Aerospace, Berlin, Germany, or model TGW-4570–4 from Telonics, Mesa, Arizona) from which 14 (9 males and 5 females) were captured on the RAFH range (18 bear-years; 2014–2019) and 26 (15 males and 11 females) on the RGH range (50 bear-years; 2012–2019; Table 1). We programmed the collars to collect locations every 4 hours during the active period of bears (April-October). We used a mixture of Telazol (1.5 mg/kg) and medetomidine (0.04 mg/kg) to immobilize black bears at capture. We collected blood samples during handling procedures to conduct stable isotope analyses (see “Trophic position of bears” section below). Wildlife handling and sample collections for live capture followed the Canadian Council on Animal Care guidelines, and all procedures were approved by the MELCCFP (CPA-FAUNE 2011039) and Université Laval (2014011, and 2018034) Animal Care Committees. As the project was conducted in partnership with the government and all captures occurred on public lands, no permit was required to access the field sites.

**Table 1:** Number of male and female black bears with telemetry data and relative trophic position in the ranges of the Rivière-aux-Feuilles and Rivière-George migratory caribou herds in northern Québec and Labrador, 2012-2019.

Year	RAFH			RGH		
	Male	Female	Total	Male	Female	Total
2012	0	0	0	4	4	8
2013	0	0	0	2	3	5
2014	1	0	1	7	4	11
2015	0	0	0	3	3	6
2016	0	0	0	5	6	11
2017	6	3	9	2	1	3
2018	3	2	5	2	4	6
2019	1	2	3	0	0	0
**Total bear-years**	11	7	18	25	25	50
**Unique IDs**	9	5	14	15	11	26

RAFH, Rivière-aux-Feuilles herd (northern Québec); RGH, Rivière-George herd (northern Québec and Labrador).

### Trophic position of bears

We conducted stable isotope analyses on the blood serum to estimate the relative trophic position of bears within the 10–15 days prior to sampling (details below). Stable isotope analysis is a robust method to estimate the relative trophic position of individuals over a certain period of time and gain insights about their foraging behavior [59,60]. Because we collected samples throughout June, we used δ15N values to assess the relative trophic position of each bear during the caribou calving period (for more details see: [37,61]). Stable isotope analyses were conducted in the Laboratoire d’Océanographie of Laval University, Québec, Canada. Isotopic analyses were performed by a continuous-flow isotope ratio mass spectrometer (Thermo Electron Delta Advantage) in the continuous-flow mode (Thermo Electron ConFlo III) and using an ECS 4010 Elemental Analyzer/ZeroBlank Autosampler (Costech Analytical Technologies). USGS40 and USGS41 were used as laboratory standards. Data are reported as parts per mil (‰). Measurement error was ± 0.1‰ for δ15N. The values of relative trophic position of bears varied between 5.37 and 8.94 for bears in the RAFH range and 3.41 and 10.63 for bears in the RGH range (S1 Fig).

### GPS data, space use and movement metrics

To determine whether bears with higher relative trophic positions performed particular movement and space use patterns to access caribou calves, we used GPS locations of female caribou and black bears during the caribou calving period. We defined the calving period for each year using first-passage time analysis to detect abrupt changes in movement patterns of migratory caribou and to identify arrival and departure dates on the calving grounds as detailed in Le Corre *et al.* (2014). Therefore, start and end dates of the calving period varied slightly between herds and among years (Table 2).

**Table 2:** Start and end dates of the calving period between 2012 and 2019 for the two migratory caribou herds in northern Québec and Labrador as defined by the method of Le Corre et al. (2014).

	RAFH		RGH	
Year	Start	End	Start	End
2012	11-Jun	06-Jul	26-May	02-Jul
2013	09-Jun	06-Jul	02-Jun	09-Jul
2014	11-Jun	04-Jul	30-May	28-Jun
2015	04-Jun	02-Jul	19-May	24-Jun
2016	09-Jun	04-Jul	23-May	28-Jun
2017	09-Jun	04-Jul	20-May	26-Jun
2018	12-Jun	08-Jul	18-May	29-Jun
2019	10-Jun	04-Jul	23-May	24-Jun

RAFH**,** Rivière-aux-Feuilles herd (northern Québec) RGH, Rivière-George herd (Labrador).

Further, we extracted movement metrics for black bears using the amt-package [62] in R-Studio version 4.4.2 (2024-10-31 ucrt) [63]. Given that the collar fix success rate was 85%, we initially resampled the telemetry data by burst, selecting only consecutive GPS locations that were exactly 4 hours apart for the analysis [62]. We then calculated mean step length and the circular mean of turning angle as indicators for speed and straightness, respectively, over the entire calving period. We only used individuals with a minimum of 40 locations per year for calculating these values as preliminary analyses revealed that estimates were less variable beyond that threshold.

To investigate if the variation in relative trophic position could be related to the space use of bears, we determined the home range size of each bear during the calving season using a kernel estimator (i.e., 95% kernelUD function in the adehabitatHR package 0.4.19) [64]. The potential access of bears to caribou neonates was assessed by examining the proximity of bears to the calving ground defined as the 95% kernel of all female caribou locations during the annual calving period for each herd separately. For each bear location, we calculated the minimal distance (m) to the closest border of the calving ground. When a bear was located within the boundaries of the calving ground, we set the distance to 0. Furthermore, we assessed the proportional overlap of the bears’ utilized ranges (95% Kernel) with the caribou calving grounds using the kerneloverlaphr function from the R package adehabitatHR [64].

We extracted habitat classes for each bear location within their ranges (95% Kernel) from a classified 2015 Landsat satellite imagery with a spatial resolution of 30 m [65]. We grouped habitat classes according to their relevance (S2 Table) in providing either forage in the form of berries (shrubland) or in increasing encounter rate with caribou neonates (caribou habitat) [19]. We defined “caribou habitat” based on habitat used by female caribou during calving which mainly consisted of tundra dominated by lichens, graminoids, and other low vegetation [66]. Furthermore, we grouped habitat classes with high amounts of shrub cover as they potentially provide bears with greater amount of forage. We then calculated the percentage of relevant habitat classes used by each based on all available locations. Because elevation was reported to increase kill rates of black bears hunting caribou calves [19], we also extracted elevation at each bear location from a digital elevation model with a 100 m resolution (Natural Resources Canada 2015). We then calculated the mean elevation across all locations of each bear during the calving season.

### Statistical analysis

To investigate the relationship between the relative trophic position of bears, movement metrics, and space use, we fitted generalized additive mixed models (GAMM) to a set of relevant candidate models (S3 Table) using R 4.4.2 (2024-10-31) with the MuMIn [67] and mgcv [68] packages. GAMMs offer the advantage of not assuming an exclusively linear relationship between the dependent and independent variables, while also allowing for the specification of each variable contribution to the prediction [67,68]. We determined the relative trophic position for each bear-year as the response variable, resulting in a total of 68 observations from 40 individual bears. We sampled some individuals more than once across the different years, and collected new blood samples at each capture. We only included a bear-year in the analysis when a corresponding blood sample was available for that specific year. We used movement metrics and space use variables as explanatory variables. To improve interpretability, we grouped models into conceptually distinct categories reflecting key ecological processes: access to caribou calving grounds, search effort, and habitat use and movement (S3 Table). In agreement with the results of previous studies in the same system [37,61], we included sex and study site in all candidate models. Before conducting the analyses, we verified the Pearson and Spearman correlations between each pair of variables and removed one of the two variables when r was > 0.6 [69,70]. We thereby excluded shrubland use which was negatively correlated with caribou habitat use (−0.92) and the minimum distance to the calving ground which was negatively correlated with the proportional overlap with the calving ground (−0.67). In both cases the correlated variables provided similar information, that is why we prioritized variables with clearer biological interpretation and stronger relevance to the behavior and habitat use of the species involved. This approach allowed us to build a model that was both parsimonious and ecologically meaningful.

We included space use and movement metrics as potential explanatory variables (all continuous predictors): range size (95% Kernel) of bears (HR), proportional overlap between bear range (95% Kernel) and caribou calving ground during the calving period (Overlap95), the percentage of bear locations recorded within suitable caribou habitat (Cari), mean elevation use (Elevation), mean step length (Step), and mean circular turning angle in degrees (Turn angle). We modeled the proportional overlap (Overlap95) as a smooth term to account for potential non-linear relationships between the overlap of bear ranges with the caribou calving ground and their relative trophic position. The smoothing term allowed to test non-linearity and to modulate a potential interaction of overlap with elevation, i.e., to test whether the effect of overlap varies across the gradient of elevation use.

Because bear capture dates varied relative to the start of the calving season, we calculated the number of days between blood sample collection, used to estimate relative trophic positions, and the onset of caribou calving. Negative values represented captures prior to the start of the calving season, while positive values indicated captures afterward, with the full range spanning from −10 to 37 (S2 Fig). We included this variable as a covariate in all models to control for the potential influence of the variation in capture dates relative to the onset of calving on the relative trophic position. We finally included bear-ID as a random effect on the intercept of all candidate models. Because bear data were not recorded over the same years for the RAFH and the RGH (see Table 1), we compared the effect size of the study site by fitting models with and without year as a random effect. Based on the identical R² values (0.32) for both models, we concluded that including year as a random effect did not improve the proportion of variance explained by the model, and therefore, decided to exclude year from the final analyses. All models assumed a Gaussian error distribution, appropriate for continuous data, and we verified this assumption through visual inspection of the residuals.

To assess model parsimony, we ranked and compared model performance using Akaike’s Information Criterion corrected for small sample size (AICc) and considered the model with the lowest AICc value as the most parsimonious [71]. We present estimates with their 95% confidence intervals (CIs) for all parameters included in the best model. We interpreted the effect of variables whose 95% CIs excluded zero.

## Results

We obtained movement metrics from GPS locations and relative trophic position from stable isotope analysis of blood serum during the calving season for 40 individual bears. This corresponds to 68 bear-years combinations with 50 bear-years from 2012–2018 on the RGH range and 18 bear-years from 2014–2019 on the RAFH range (Table 1).

During the calving period, bear home ranges (95% Kernel) averaged 1,152 ± 315 (SE) km^2^ in the RAFH range and 1,753 ± 72 (SE) km^2^ in the RGH range. Globally, the average proportion of overlap between bear ranges (95% Kernel) and caribou calving ground was 72% (Table 3). The average overlap was 37% on the RAFH and 85% on the RGH (Table 3). Of the 40 black bears monitored, 23 (58%) exhibited complete overlap and 15 (38%) showed partial overlap between their used ranges (95% Kernel) and the caribou calving ground during the calving season. Overlap was more frequent among bears on the RGH range, where all individuals (26/26) overlapped to some extent and 77% (20/26) showed complete overlap. In contrast, 50% (7/14) of bears on the RAFH range showed no overlap, and only 21% (3/14) exhibited complete overlap with the calving ground.

**Table 3:** Average proportional overlap of ranges (95% Kernel) of black bears with the caribou calving grounds from the Rivière-aux-Feuilles herd (2014, 2017-2019) and Rivière-George herd (2012-2019) with number of bear-years and bear-ID in different categories of overlap.

	Total		RAFH			RGH		
Estimator	Overlap Ø^1^	Overlap Category	Bear-year	Bear-ID	Overlap Ø^2^	Bear-year	Bear-ID	Overlap Ø^2^
Utilized range (95% Kernel)	72%	complete	4	3	37%	34	20	85%
		partial	6	5		13	10	
		null	8	7		3	3	

Ø^1^, Average proportional overlap including all bear-year *n* = 68; Ø^2^, Average proportional overlap per study site; RAFH, Rivière-aux-Feuilles migratory caribou herd (northern Québec) bear-years *n* = 18 and bear ID *n* = 14; RGH, Rivière-George migratory caribou herd (Labrador) bear-years *n* = 50 and bear ID *n* = 26; The different categories of overlap were defined as complete = 1, partial = 0.01–0.99 and null = 0.

Model selection indicated that the most parsimonious model examining the relationship between the relative trophic position of black bears and space use included use of suitable caribou habitat by bear, the proportional overlap (95% Kernel) between bear ranges and the caribou calving ground, the difference in days between capture date and the start of calving season, sex and study site (S4 Table).

**Fig 4:**
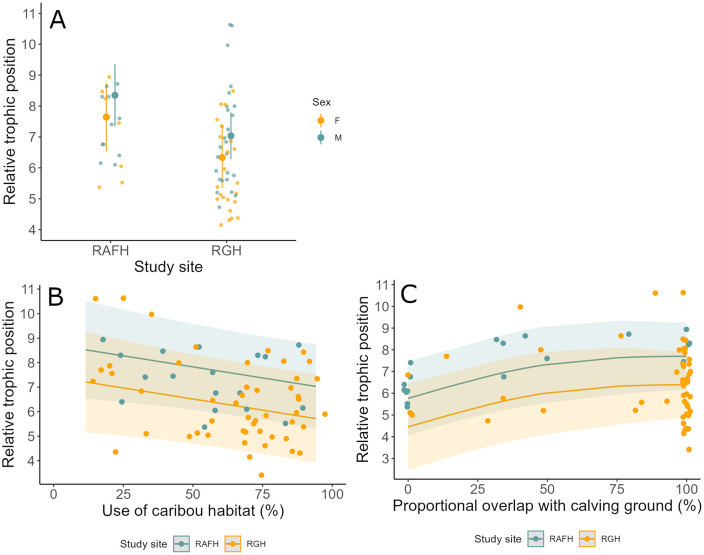
Effects of study site, use of caribou habitat by black bears, and proportional overlap of bear ranges (95% Kernel) with the caribou calving ground on the relative trophic position of black bears in northern Québec and Labrador 2012-2019, based on a generalized additive mixed model (GAMM). **(A)** Differences in relative trophic position between black bears using the Rivière-aux-Feuilles (RAFH) and Rivière-George (RGH) calving grounds. **(B)** Effect of the use of caribou habitat by black bears on their relative trophic position. **(C)** Effect of the proportional overlap of bear ranges (Kernel 95%) with the caribou calving ground on the relative trophic position of black bears.

Bears foraging on the RAFH range had a higher relative trophic position than bears foraging on the RGH range (Fig 4A). Relative trophic position did not significantly differ between sexes. The use of caribou habitat by a bear was negatively correlated with its relative trophic position (Fig 4B). Furthermore, we found that bears with more pronounced overlap of their ranges (95% Kernel) with the caribou calving grounds exhibited higher relative trophic positions (Fig 4C). We found substantial variation in relative trophic position among individual bears, as captured by the random effect of bear ID, highlighting individual differences beyond the fixed effects in the model (Table 4). Moreover, relative trophic position showed no significant relationship with the difference in capture dates relative to calving, or with any other movement or space use metrics (Table 4).

**Table 4:** Results of the most parsimonious generalized additive mixed model based on Akaike’s Information Criterion adjusted for small sample size (AICc) to explain variation in relative trophic position of black bears in northern Québec and Labrador during caribou calving seasons 2012-2019 (n = 68 bear-years). Estimates whose 95% confidence intervals do not include zero are highlighted in bold.

Predictor		Estimate	SE	Cl 95%	
(Intercept)		8.52	0.67	7.17–9.88	
Use of caribou habitat		**−0.02**	**0.01**	**−0.03 – −0.00**	
Difference in days between capture date and start of calving season		−0.01	0.02	−0.04–0.03	
Sex [Male]		0.70	0.42	−0.14–1.55	
Study site [Rivière-George herd]		**−1.31**	**0.50**	**−2.33 – −0.30**	
Smooth term		**edf**	**F-statistic**	**p-value**	
Proportional overlap (95% Kernel) with calving ground		**1.79**	**6.63**	**0.003**	
Random effect			SD	ICC	τ₀₀
Bear ID *n* = 40	16.10	0.92	0.75	0.33	0.57
Observations	68				
R^2^	0.54				

Cl 95%, 95% confidence interval; edf, Effective degrees of freedom, SD, Standard deviation; ICC, Intraclass correlation coefficient τ₀₀, Variance.

## Discussion

The joint analysis of movement and diet data of large predator species is challenging because several intrinsic and extrinsic factors not directly related to foraging and food intake may drive movements and space use patterns of individuals. By integrating both movement and diet data, our study provides innovative and novel insights in how black bears respond to caribou calves as a pulsed resource. We found that variation in the relative trophic position was influenced by both environmental and individual factors. We corroborated the findings of previous studies [61] indicating that the study site significantly influenced the relative trophic position of black bears in northern Québec and Labrador. Bears from the RAFH had a higher relative trophic position than those from the RGH during the calving season. This may be due to higher availability of alternative plant-based food resources at the Labrador site. Importantly, we discovered that bears with higher relative trophic positions exhibited greater spatial overlap with caribou calving grounds. Contrary to our initial prediction, we found, however, that bears utilizing habitats considered most suitable for caribou during calving (caribou habitat) had lower relative trophic positions. These results suggest that foraging strategies in black bears may operate at finer spatial or temporal scales than those captured by our metrics, or that trophic signals integrate a wider range of foraging behaviors, including scavenging.

Differences in relative trophic position among bears from the RAFH and RGH ranges are most likely attributable to variations in resource availability. Given the wide difference in caribou abundance between herds, the difference in relative trophic position between bears from the two study sites may reflect the relative availability of caribou calves on the calving grounds. Caribou availability is expected to be higher in northern Québec than in Labrador, as the RAFH had higher population estimates than the RGH during our study period. During our study, the RAFH population was estimated at around 200,000 individuals, while the RGH was estimated at less than 27,000 individuals in 2012, declining to a low abundance of about 5,500 caribou in 2018. Although plant productivity is generally low in the subarctic tundra, it is likely higher for bears in the RGH range than in the RAFH range due to factors such as climate differences and the typically longer snow cover in northern Quebec than in Labrador [72]. Additionally, the relative trophic positions of bears on the RAFH range were not only higher but showed less variation over the active period of bears [61] compared to bears on the RGH, suggesting that they rely more on animal matter to gain mass throughout their active period than on vegetation. Although a bear would not pass up the opportunity to consume animal protein, when alternative food sources are available, it would primarily forage on vegetation and consume animal matter only when encountered incidentally. Bonin et al. [61] also showed that remains of caribou calves were more present in feces of black bears in northern Québec near the calving ground of the RAFH compared to bears foraging on or in adjacent areas of the RGH calving ground. Therefore, black bears from the RAFH should be more likely to respond to the caribou calving season and take advantage of the presence of a pulse of high-protein food source than bears foraging on the RGH range. Investigating movement and space use patterns and their seasonal changes at finer scales might reveal spatio-temporal relationships in the use of caribou by black bears.

Dietary differences between sexes are common [73]. In polygynous species like black bears, males are typically larger than females [74] and have higher metabolic demands [26,75] resulting in increased movement rates and larger home range sizes [76,77]. These more mobile behaviors could be related to increased search effort for animal prey [27,78] explaining higher relative trophic positions. Both sexes can adjust their movements according to seasons and food availability, but males usually occupy larger areas than females [35,79] to fulfill their energetic needs. However, a variety of factors such as mating and reproductive status [80] can also affect movements and space use in distinct manners for each sex. Female bears with cubs, for example, may adjust space use to avoid encounters with males that consequently may result in the use of food sources of lower quality [81]. Given that the mating season of black bears takes place from mid-May to August with a peak around mid-June to mid-July [82] overlapping our study period, the movements of black bears might also reflect the search for potential mates [77,83,84] rather than solely their foraging tactic. This could explain why movements metrics were generally poorly related to the relative trophic position of black bears in northern Québec and Labrador during early spring and summer.

Investigating the differences in movement tactics before and after the calving season could help to better identify the adjustments of black bear behaviors in relation to the caribou calving season. Coupling such an approach with repeated measurements of relative trophic positions of bears could foster the study of foraging ecology of black bears. We hypothesized that bears utilizing areas with higher densities of female caribou, such as the calving ground or caribou-favored habitats during the calving season, would be more likely to encounter caribou and, consequently, to use them as a food source. This should be the case because caribou neonates occur as a pulse and are more vulnerable to predation than adults [40,41]. We found evidence that bears whose ranges overlapped more extensively with the caribou calving grounds had higher relative trophic positions than bears whose ranges overlapped only partially or not at all with caribou calving grounds. Because the relative trophic position did not relate to a bear’s range size during the caribou calving period, we suggest that bears that were residing exclusively on the calving grounds during the caribou calving season should have exploited caribou more intensively than bears using a large area including part of the calving grounds. Bears with partial overlap with the calving grounds foraged outside or at the periphery of the calving ground where they likely experienced lower densities of caribou. Although caribou may still be available to those bears, it may be more efficient for them to seek other resources which might lead to lower relative trophic positions. Surprisingly, we found that the relative trophic position of black bears was negatively related to the use of habitats that female caribou preferred during the calving season. We suggest that at calving, female caribou may seek out safer habitats, potentially providing better visibility for detecting predators early or allowing for easier escape from approaching threats. Moreover, the negative correlation between the use of caribou habitat and the use of shrubland by bears suggests that the relative trophic position was higher for individuals using areas with greater shrub cover. Bears foraging in areas with dense shrub cover may experience higher hunting success due to surprise encounters with caribou or because dense vegetation limits the escape options for caribou. The presence of habitat features that could improve hunting success for bears at a finer scale or increase caribou vulnerability may be more significant in predicting caribou consumption by bears than simply the extent of their spatial overlap [19,85,86]. To further investigate the foraging tactics of bears in relation to caribou calves, we would need to map the distribution of calves within the calving area and assess whether fine-scale habitat selection by bears is linked to the local availability of caribou calves. For instance, identifying stalking cover, such as vegetation or terrain roughness, could provide insights into how enhanced hunting success translates into higher relative trophic positions [19,87].

Relative trophic position may not be linked to movement metrics because black bears are fundamentally opportunistic, accessing food items sequentially as they move. While we expected the relative trophic position to reflect whether an individual’s diet mainly consisted of vegetation or animal proteins over a given period, opportunistic foraging tactics may result in relative trophic position values closer to the average. A mainly carnivorous or herbivorous diet are the two extremes within the foraging range of bears and might therefore be rare and difficult to detect. Most values of relative trophic position are, therefore, likely to at least partly result from opportunistic foraging. Across its distribution range in eastern North America, a large part of the black bear diet consists of plants such as grasses and berries during the caribou calving season [37–39]. In addition, a higher relative trophic position, resulting from greater meat consumption, does not imply active hunting behavior, nor the sole use of caribou neonates as prey. Although black bears residing on the calving grounds might have higher access to caribou neonates, bears may also access animal proteins by scavenging on caribou carrion found on the caribou migration routes and the calving grounds (e.g., stillborn calves or placenta remains, carcasses of dead caribou). As bears are known to be generalist omnivores potentially feeding on several alternative animal food sources such as fish, eggs, or small mammals [38,88,89], they are not dependent on the consumption of calves or the access to calving grounds to increase their intake in animal proteins and consequently their relative trophic position.

Although we did not find evidence of a correlation between movement metrics and relative trophic position of bears, it is nevertheless known that individuals can specialize on certain food sources [26]. Specialization on a mainly carnivorous diet might be rare within black bear populations but is most likely to occur in northern environments [37]. Therefore, some black bears may display unique movement patterns related to their food intake, which could be challenging to detect, particularly if these behavioral adjustments occur at a very fine temporal or spatial scale. Investigating foraging behavior at the individual level and at a finer scale, for both the predator and the prey, might therefore be relevant to better understand black bear foraging tactics in relation to a pulsed resource within a subarctic environment.

## Supporting information

S1 Fig Frequency distributions of the relative trophic positions for all black bears included in the analyses (A), for bear-years on the ranges of the Rivière-aux-Feuilles (RAFH) (B; n = 18) and the Rivière-George (RGH) migratory caribou herds (C; n = 50). (TIFF)

S2 Fig Capture dates of black bears (black points) on the ranges of the Rivière-aux-Feuilles (RAFH) and the Rivière-George (RGH) migratory caribou herds in relation to the caribou calving season (grey line), as defined by the method of Le Corre et al. (2014), between 2012 and 2019. (TIFF)

S1 Table Number of female caribou monitored during the calving periods in the ranges of the Rivière-aux-Feuilles and the Rivière-George migratory caribou herds between 2012–2019. We estimated the size of their annual calving ground (km^2^) using telemetry data with a 95% Kernel estimator.
(PDF)

S2 Table Landsat habitat classes with their description from the North American Land Change Monitoring System (2020), that we used to define two habitat types (shrubland and caribou habitat) likely to provide black bears with forage in the form of berries or increased encounter rates with caribou, respectively. (PDF)

S3 Table Candidate models formulated to explain variation in relative trophic position in black bears on the Rivière-aux-Feuilles (RAFH) and Rivière-George (RGH) ranges in northern Québec and Labrador. We included mean step length (Step), mean turning angle (Turn angle), caribou habitat use (Cari), elevation use (Elevation), proportional overlap with the caribou calving ground (Overlap95) and home range size (HR) as explanatory variables. We controlled for the effects of sex (Sex) and study area (Study_site) by forcing these variables in all models. We categorized the models into conceptually distinct groups based on key ecological processes: access to caribou calving grounds, search effort, and habitat use and movement. Bear ID was included in all models as a random factor.
(DOCX)

S4 Table Model selection results for candidate models testing the effects of trophic position of black bears on the Rivière-aux-Feuilles range and Rivière-George ranges. We present the number of parameters (k), the Akaike’s Information Criterion for small sample sizes (AICc), the AICc difference from the top model (∆AICc), and the log-likelihood of the model (LL). We considered the most parsimonious model as the one with the lowest AICc.
(DOCX)
