# Supplementary figures and images for: Combining space use with diet data to investigate foraging tactics of black bears in response to the pulsed availability of migratory caribou calves
Source: PLoS One. 2026 Apr 3;21(4):e0346054. doi: 10.1371/journal.pone.0346054 (PMC13048383; doi:10.1371/journal.pone.0346054)

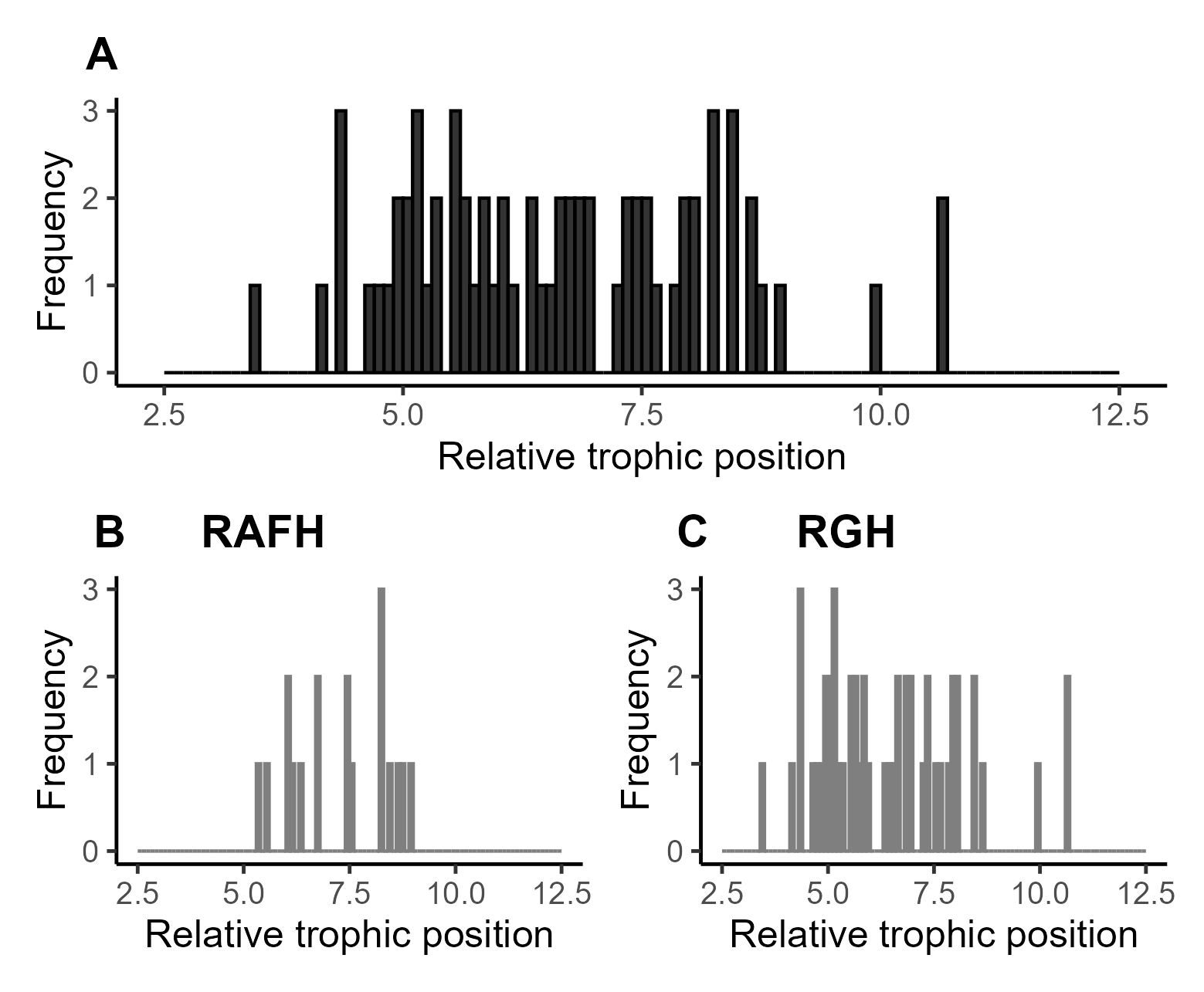

Supplement: S1 Fig — (TIFF) [file pone.0346054.s001.tiff]

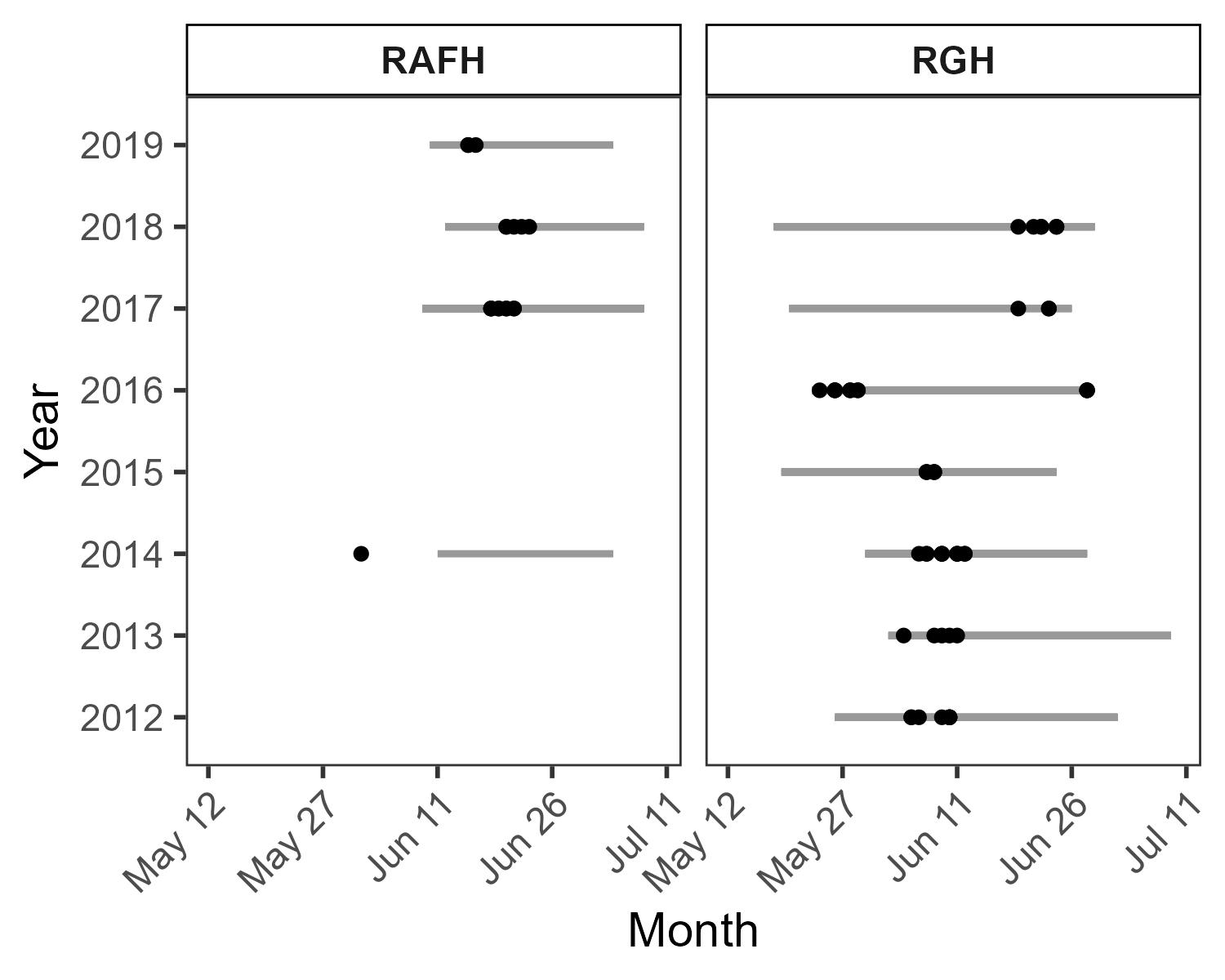

Supplement: S2 Fig — (TIFF) [file pone.0346054.s002.tiff]
