# Supplementary material for: Combining space use with diet data to investigate foraging tactics of black bears in response to the pulsed availability of migratory caribou calves
Source: PLoS One. 2026 Apr 3;21(4):e0346054. doi: 10.1371/journal.pone.0346054 (PMC13048383; doi:10.1371/journal.pone.0346054)
Supplement: S1 Table — We estimated the size of their annual calving ground (km2) using telemetry data with a 95% Kernel estimator. (PDF) [file pone.0346054.s003.pdf]

**Year**	**RAFH**		**RGH**	
	Nr females	Calving range (km^2)	Nr. Females	Calving range (km^2)
**2012**	72	45167	73	47622
**2013**	101	52842	69	6794
**2014**	92	79397	73	7122
**2015**	67	64739	57	7518
**2016**	90	84750	52	6862
**2017**	83	54195	56	13118
**2018**	70	85967	53	8551
**2019**	76	83358	44	22138
**Average**	81	68802	60	14966

RAFH, = Rivière-aux-Feuilles migratory caribou herd,; RGH, = Rivière-George migratory caribou herd.
