# Supplementary material for: Combining space use with diet data to investigate foraging tactics of black bears in response to the pulsed availability of migratory caribou calves
Source: PLoS One. 2026 Apr 3;21(4):e0346054. doi: 10.1371/journal.pone.0346054 (PMC13048383; doi:10.1371/journal.pone.0346054)
Supplement: S2 Table — (PDF) [file pone.0346054.s004.pdf]

**Landsat habitat-class**	**Description**	**Habitat type**
1	Temperate or sub-polar needleleaf forest	
2	Sub-polar taiga needleleaf forest	
5	Temperate or sub-polar broadleaf deciduous forest	
6	Mixed forest	
8	Temperate or sub-polar shrubland	shrubland
11	Sub-polar or polar shrubland-lichen-moss	
12	Sub-polar or polar grassland-lichen-moss	caribou-habitat
13	Sub-polar or polar barren-lichen-moss	
18	Water	
14	Wetland	
