# Supplementary material for: Combining space use with diet data to investigate foraging tactics of black bears in response to the pulsed availability of migratory caribou calves
Source: PLoS One. 2026 Apr 3;21(4):e0346054. doi: 10.1371/journal.pone.0346054 (PMC13048383; doi:10.1371/journal.pone.0346054)
Supplement: S3 Table — We included mean step length (Step), mean turning angle (Turn angle), caribou habitat use (Cari), elevation use (Elevation), proportional overlap with the caribou calving ground (Overlap95) and home range size (HR) as explanatory variables. We controlled for the effects of sex (Sex) and study area (Study_site) by forcing these variables in all models. We categorized the models into conceptually distinct groups based on key ecological processes: access to caribou calving grounds, search effort, and habitat use and movement. Bear ID was included in all models as a random factor. (DOCX) [file pone.0346054.s005.docx]

| **Model** | **Independent Variables Conceptual group** |
| --- | --- |
| Mod1 | Null |
|  | **Control** |
| Mod2 | Diff_in_days + Sex + Study_site |
|  | **Single effects** |
| Mod3 | HR + Diff_in_days + Sex + Study_site |
| Mod4 | Step + Diff_in_days + Sex+ Study_site |
| Mod5 | Turn angle + Diff_in_days + Sex+ Study_site |
| Mod6 | Cari + Diff_in_days + Sex+ Study_site |
| Mod7 | Elevation + Diff_in_days + Sex+ Study_site |
| Mod8 | Overlap95 + Diff_in_days + Sex+ Study_site |
|  | **Access to caribou calving ground** |
| Mod9 | Overlap95 + Turn angle + Diff_in_days + Sex+ Study_site |
| Mod10 | Overlap95 + Step + Diff_in_days + Sex+ Study_site |
| Mod11 | Overlap95 + (Turn angle * Step) + Diff_in_days + Sex+ Study_site |
| Mod12 | Overlap95 + Cari + Diff_in_days + Sex+ Study_site |
| Mod13 | Overlap95 + Elevation + Diff_in_days + Sex + Study_site |
| Mod14 | Overlap95 + (Elevation * Cari) + Diff_in_days + Sex + Study_site |
| Mod15 | Overlap95 + HR + Diff_in_days + Sex + Study_site |
| Mod16 | Overlap95: Elevation + Diff_in_days + Sex + Study_site |
|  | **Search effort** |
| Mod17 | HR + Step + Diff_in_days + Sex + Study_site |
| Mod18 | HR + Turn angle + Diff_in_days + Sex + Study_site |
| Mod19 | HR + (Turn angle * Step) + Diff_in_days + Sex + Study_site |
| Mod20 | HR +Cari+ Step + Diff_in_days + Sex + Study_site |
| Mod21 | HR +cari+ Turn angle + Diff_in_days + Sex + Study_site |
| Mod22 | HR + (Cari * Elevation) + Diff_in_days + Sex + Study_site |
|  | **Habitat use and movement** |
| Mod23 | Elevation + Step + Diff_in_days + Sex + Study_site |
| Mod24 | Elevation + Turn angle + Diff_in_days + Sex + Study_site |
| Mod25 | Elevation + (Turn angle * Step) + Diff_in_days + Sex + Study_site |
| Mod26 | Cari + (Turn angle * Step) + Diff_in_days + Sex + Study_site |
| Mod27 | Elevation + Cari+ (Turn angle * Step) + Diff_in_days + Sex + Study_site |
| Mod28 | Elevation + Cari+ HR+ (Turn angle * Step) + Diff_in_days + Sex + Study_site |
| Full model | Elevation + Cari+ HR + Turn angle + Step + Overlap95 + Diff_in_days + Sex + Study_site |
