# Supplementary material for: Combining space use with diet data to investigate foraging tactics of black bears in response to the pulsed availability of migratory caribou calves
Source: PLoS One. 2026 Apr 3;21(4):e0346054. doi: 10.1371/journal.pone.0346054 (PMC13048383; doi:10.1371/journal.pone.0346054)
Supplement: S4 Table — We present the number of parameters (k), the Akaike’s Information Criterion for small sample sizes (AICc), the AICc difference from the top model (∆AICc), and the log-likelihood of the model (LL). We considered the most parsimonious model as the one with the lowest AICc. (DOCX) [file pone.0346054.s006.docx]

| **Model** | **k** | **AICc** | **∆AICc** | **LL** |
| --- | --- | --- | --- | --- |
| Mod12 | 22 | 247.8 | 0.00 | -89.57 |
| Mod10 | 24 | 250.7 | 2.97 | -86.93 |
| Mod9 | 23 | 251.4 | 3.61 | -88.79 |
| Mod8 | 23 | 251.9 | 4.10 | -87.98 |
| Mod11 | 25 | 252.6 | 4.79 | -84.31 |
| Mod13 | 24 | 253.3 | 5.50 | -86.44 |
| Mod5 | 19 | 256.0 | 8.20 | -99.33 |
| Mod6 | 20 | 256.1 | 8.30 | -98.84 |
| Mod1 | 21 | 256.1 | 8.35 | -96.89 |
| Mod26 | 22 | 256.8 | 9.00 | -93.59 |
| Mod14 | 26 | 256.9 | 9.17 | -83.95 |
| Mod2 | 20 | 257.2 | 9.40 | -97.87 |
| Mod16 | 25 | 257.5 | 9.79 | -87.49 |
| Mod24 | 20 | 258.1 | 10.36 | -98.48 |
| Mod4 | 21 | 258.2 | 10.47 | -97.73 |
| Mod25 | 23 | 258.3 | 10.58 | -91.93 |
| Mod7 | 21 | 259.2 | 11.46 | -97.48 |
| Mod27 | 23 | 260.7 | 12.93 | -92.44 |
| Mod23 | 22 | 260.8 | 12.99 | -96.95 |
| Mod29 | 5 | 302.5 | 54.74 | -144.67 |
| Mod28 | 4 | 304.3 | 56.55 | -146.67 |
| Mod19 | 4 | 348.2 | 100.48 | -196.80 |
| Mod20 | 3 | 348.5 | 100.75 | -171.07 |
| Mod17 | 3 | 348.6 | 100.82 | -171.10 |
| Mod22 | 3 | 352.3 | 104.54 | -171.83 |
| Mod3 | 1 | 451.5 | 203.70 | -223.64 |
| Mod15 | 1 | 451.5 | 203.70 | -223.64 |
| Mod18 | 1 | 451.5 | 203.70 | -223.64 |
| Mod21 | 1 | 451.5 | 203.70 | -223.64 |
